# Quality assessment and optimization of purified protein samples: why and how?

**DOI:** 10.1186/s12934-014-0180-6

**Published:** 2014-12-30

**Authors:** Bertrand Raynal, Pascal Lenormand, Bruno Baron, Sylviane Hoos, Patrick England

**Affiliations:** Institut Pasteur, Biophysics of Macromolecules and their Interactions, 25 rue du Docteur Roux, 75724 Paris Cedex 15, France; CNRS-UMR3528, Institut Pasteur, Departement of Structural Biology and Chemistry, Paris, France

**Keywords:** Recombinant protein, Therapeutic, Diagnosis, Structural biology, Electrophoresis, Mass spectrometry, UV/visible spectroscopy, Light scattering, Size-exclusion chromatography, Surface plasmon resonance, Formulation, Comparability

## Abstract

Purified protein quality control is the final and critical check-point of any protein production process. Unfortunately, it is too often overlooked and performed hastily, resulting in irreproducible and misleading observations in downstream applications. In this review, we aim at proposing a simple-to-follow workflow based on an ensemble of widely available physico-chemical technologies, to assess sequentially the essential properties of any protein sample: purity and integrity, homogeneity and activity. Approaches are then suggested to optimize the homogeneity, time-stability and storage conditions of purified protein preparations, as well as methods to rapidly evaluate their reproducibility and lot-to-lot consistency.

## Introduction

In recent years, purified proteins have more and more frequently been used for diagnostic and therapeutic applications [1-3]. Purified proteins are also widely used as reagents for downstream in depth biophysical and structural characterization studies: these are sample- and time-consuming, generally requiring long set-up phases and sometimes depending on (limited) accessibility to large instrumentation such as synchrotrons.

Unfortunately, scientists (especially in the academic environment) frequently want to rush to the final application, considering biochemical analysis of proteins as either trivial or a superfluous bother. Very often, the implications of such a regretful attitude are irreproducible, dubious and misleading results, and unfortunately sometimes lead to failure at more or less advanced stages (including clinical trials [4]), with potentially severe consequences. This is even more the case nowadays, when recombinant production of challenging proteins such as integral membrane proteins or heavily modified (glycosylated, …) proteins is being attempted on an ever more widespread scale.

The correct interpretation of many biophysical/structural characterization experiments relies on the assumption that:

1. the protein samples are pure and homogeneous.
2. their concentration is assessed precisely.
3. all of the protein is solubilized and in a natively active state.

Our experience as a core facility dealing with several dozens of different projects every year is that quality control considerations are much too often overlooked or taken for granted by facility users and the scientific community at large. However, those who assess and optimize carefully the quality of their protein preparations significantly increase their chances of success in subsequent experiments.

Purified protein quality control has already been the object of several general reviews [5-7]. Attempts have also been made to define a set of “minimal quality criteria” that should be fulfilled by any purified recombinant protein prior to publication, especially among the “Minimal Information for Protein Functionality Evaluation” (MIPFE) consortium [8-10]. In this review, we wish to go one step further and provide a concise overview of a sequence of simple-to-follow physico-chemical approaches that should be accessible to the vast majority of investigators. Most of the methodologies that are proposed can be found in classical biochemistry or structural biology laboratories, and in the majority of institutional protein science core facilities. Many of the methods and techniques mentioned here are well known, maybe too well, but clearly need to be reappraised in university curricula and laboratory practice: indeed knowledge about them is generally (and inappropriately) regarded as obvious, but very often it is in reality very sketchy, sometimes unfortunately resulting in gross blunders. Hopefully, this review will help providing more robustness to the production of efficient and reliable protein samples within a large scientific community.

## Protein quality control methodological work-flow

### Initial Sample assessment

#### Purity and integrity

##### Electrophoresis

**Figure 1:**
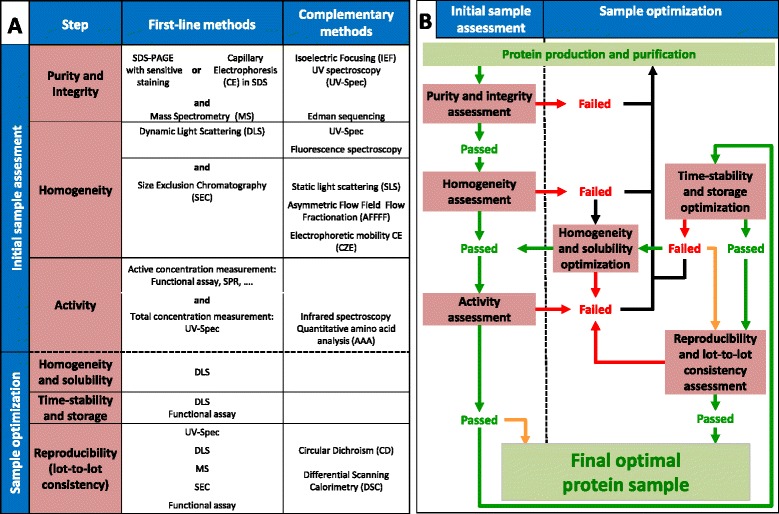
**Experimental protein quality control methodological work-flow. A)** The properties (purity & integrity, homogeneity, activity) to be assessed for each new protein sample are listed on the upper left. First-line methods are essential and should be used systematically for a full quality control assessment. Complementary methods can be added depending on the protein sample peculiarities and quality control requirements. Similarly, methods for sample optimization monitoring are grouped below in two categories: first-line and complementary. **B)** The work flow has to be followed step-by-step starting with the “protein production and purification” green box. For each step, achievement of quality criteria is indicated by a green arrow (passed) while failure is indicated by a red arrow (failed). In case of failure, process optimization has to be carried out as indicated by black arrows. Initial sample assessment is sufficient if a sample is only produced once and used directly without storage (orange arrow at the bottom left). In contrast, if samples have to be stored for an undetermined period of time and produced several times, the sample optimization part of the work-flow should be performed thoroughly. If no appropriate storage conditions can be found, one should work only with fresh preparations (orange arrow on the right).

Prior to any downstream experiment, purity and integrity are the very first qualities that need to be assessed for any protein sample (Figure 1B). This is routinely achieved by Sodium Dodecyl Sulfate Polyacrylamide Gel Electrophoresis (SDS–PAGE). This technique, associated with Coomassie blue staining, can detect bands containing as little as 100 ng of protein in a simple and relatively rapid manner (just a few hours) [11]. After reduction and denaturation by SDS, proteins migrate in the gel according to their molecular mass, allowing to detect potential contaminants, proteolysis events, etc. However, many low amount impurities and degradation products can go unnoticed, especially in low concentration samples or during optimization phases in which minute aliquots are analysed.

Two higher sensitivity colorimetric staining methods can be used either directly after electrophoresis or coupled to Coomassie blue staining: zinc-reverse staining [12] and silver staining [13]. These can detect as low as 10 ng and 1 ng protein bands respectively. Zinc-reverse staining (also known as negative staining) uses imidazole and zinc salts for protein detection in electrophoresis gels [12]. It is based on the precipitation of zinc imidazole in the gel, except in the zones where proteins are located. When zinc-reverse staining is applied on a Coomassie blue stained gel, previously undetected bands can be spotted [14]. This technique is rapid, simple, cheap and reproducible, and is compatible with mass spectrometry (MS) [15]. On the other hand, silver staining is based on the binding of silver ions to the proteins followed by reduction to free silver, sensitization and enhancement [13]. If used as a second staining, it is essential to fix the proteins in the gel with acidic alcohol prior to initial Coomassie blue staining [16]. Two drawbacks of this technique are that proteins are differentially sensitive to silver staining and the process may irreversibly modify them preventing further analysis. In particular glutaraldehyde, which is generally used during the sensitization step, may interfere with protein analysis by MS due to the introduction of covalent cross-links [17]. To circumvent this problem, a glutaraldehyde-free modified silver-staining protocol has been developed, which is compatible with both matrix-assisted laser desorption/ionization (MALDI) and electrospray ionization-MS [17].

Several fluorescent dyes such as Nile red, ruthenium(II) tris(bathophenantroline disulfonate) (RuBPS), SyPro and Epicocconone, can also be used to reveal a few ng of proteins in gels [18-20]. CyDyes can even reveal amounts of protein lower than one nanogram but have the inconvenience of requiring to be incorporated before gel electrophoresis [20]. Apart from Nile red, these staining methods are compatible with subsequent MS analysis. However, their major disadvantage is that they require a fluorescence imager for visualization and that they are significantly more expensive than classical colorimetric dyes.

Different alternatives (or additions) to SDS-PAGE exist to further separate and distinguish the protein of interest from closely related undesired subproducts or contaminants. One of them is isoelectric focusing (IEF), which separates non-denatured proteins based on their isoelectric point, most often on gel strips. This allows to resolve proteins of very similar mass, notably unmodified and small molecular mass post-translationnally modified (e.g. phosphorylated) variants of a same protein. IEF is often used upstream of SDS-PAGE in so-called 2D gel electrophoresis [21] .

Capillary electrophoresis (CE) is another useful alternative, with the advantage of superior separation efficiency, small sample consumption, short analysis time and automatability. CE separates proteins, with or without prior denaturation, in slab gels or microfluidic channels, according to a variety of properties, including their molecular mass (SDS-CGE), their isoelectric point (CIEF) or their electrophoretic mobility (CZE) [22]. Interestingly, CE can readily be coupled on line with MS [23].

##### UV-visible spectroscopy

UV-visible spectroscopy is most often used for protein concentration measurements (see Total protein concentration determination section). However, it is also a very convenient tool for the detection of non-protein contaminants, as long as the protein of interest contains aromatic residues and the absorbance is monitored over a large range (at least 240 – 350 nm). In particular, undesired nucleic acid contaminants can be spotted as bumps at 260 nm, resulting in a high 260/280 nm absorbance ratio (which should be close to 0.57 for a non-contaminated protein sample [24]). On the other hand, reducing agents (especially DTT) alter the symmetry of the 280 nm absorbance peak by increasing the absorbance at 250 nm and below [25,26].

##### Mass spectrometry

It is essential to verify the integrity of the protein of interest beyond SDS-PAGE, especially when setting-up a new production/purification protocol, as low level proteolysis events (affecting just a few amino acids) and undesired modifications may go unnoticed in electrophoresis. The method of choice for detailed analysis of protein primary structure is MS, as it can provide molecular mass with 0.01% accuracy for peptides or proteins with masses up to 500,000 Da using only a few picomoles of sample [27]. The presence of undesired proteolytic events and chemical alterations can be readily detected by comparing the difference between the observed and the expected mass of the protein. Furthermore MS can provide detailed information about the presence of desired post-translational modifications (phosphorylations, acetylations, ubiquitinations, glycosylations, …) [28]. Overall the convenience and precision of MS measurements is such that they should be considered as routine to ensure the integrity and overall state of modification of the peptide or protein of interest.

MS-based methods, such as MALDI in-source decay [29], are progressively replacing traditional protein sequencing by Edman degradation [30]. However, N-terminal Edman sequencing is still of relevance in several cases, for instance when one wishes to verify easily and specifically the N-terminal boundary of the protein of interest, or when highly accurate masses cannot be obtained by MS because of the size of the protein or the presence of certain post-translational modifications [31].

One may also wish to further characterize the degradation products or contaminants detected by electrophoresis, as determining their origin may give clues about how to avoid them from occurring. Proteins extracted from gel bands can be digested and analysed by MS [32]. Identification can be achieved by peptide mass finger-printing, as the precise peptide pattern that results from the digestion of a protein by a sequence-specific protease (like trypsin) is unique for each protein and can be matched by protein-sequence database search [32]. Usually MALDI time-of-flight (TOF) spectrometers are used for this type of analysis because of their speed, mass accuracy and sensitivity. Typically, proteins detected by Coomassie blue or negative staining can be identified.

#### Homogeneity

##### Dynamic light scattering

Once the purity and integrity of the protein sample has been assessed, one has to ensure it is homogeneous (Figure 1). Dynamic light scattering (DLS), because of its rapidity and low sample consumption, is a very convenient method to determine simultaneously the monodispersity of the species of interest and the presence of soluble high-order assemblies and aggregates [33]. DLS measures Brownian motion, which is related to the size of the particles. The velocity of the Brownian motion is defined by a translational diffusion coefficient that can be used to calculate the hydrodynamic radius, i.e. the radius of the sphere that would diffuse with the same rate as the molecule of interest. This is done by measuring, with an autocorrelator, the rate at which the intensity of the light scattered by the sample fluctuates. As a 3 nm radius particle scatters 1 million times less light than a 60 nm one, DLS is the method of choice to detect small quantities of aggregates in a sample [34]. A few percent of large aggregates may even swamp the scattered light coming from small particles. It is important to notice that large particles may also originate from poor buffer preparation (all protein purification and storage buffers should systematically be filtered prior to use). Autocorrelation functions can be mathematically resolved using a variety of algorithms, developed either by instrument manufacturers or academic researchers (for instance Sedfit [35]). However, the robustness of these mathematical solutions is fairly poor. Moreover, a precise quantification of each individual species is difficult and the resolution of DLS does not allow to resolve close quaternary structures (for instance monomers from dimers and small-order oligomers). Overall, DLS is such an easy and convenient technique that the danger of over-interpreting its quantitative results is high [34]. However, the technique is very well adapted for qualitative studies (which are the focus of this review) and can be performed over time and/or at different temperatures in order to test the stability of the protein preparation in different buffers (see Optimization of homogeneity and solubility section).

##### UV-visible and fluorescence spectroscopies

Although less sensitive than DLS, UV-visible spectroscopy is also of use to detect the presence of large particles (with a hydrodynamic radius higher than 200 nm) in a protein preparation. This can be done by monitoring the absorbance signal above 320 nm, where aggregate-free protein samples are not supposed to absorb light, and the signal can be attributed exclusively to the scattering of light by large aggregates present in the sample. This simple measurement can quickly provide qualitative information about the sample of interest. If the UV visible signal is used for concentration measurement, the contribution of scattering to the overall absorbance can be deduced by tracing a log-log plot of absorbance versus wavelength in the 320–350 nm region. This can then be extrapolated to the rest of the spectrum [26,36].

One interesting alternative to UV-visible spectroscopy is fluorescence spectroscopy [37]. After excitation at 280 nm, the fluorescence emission signal is measured at 280 nm and 340 nm, corresponding respectively to light scattering and intrinsic protein fluorescence. The ratio of the intensities at 280 nm and 340 nm (I_280_/I_340_) is concentration independent and purely related to the degree of aggregation of the sample. This ratio, also called aggregation index (AI), should be close to zero for aggregate-free protein preparations and can attain high values (>1) when significant aggregation occurs.

##### Size-exclusion chromatography

As already stressed above, DLS does not have the sufficient resolution to correctly assess whether a protein sample is heterogeneous in terms of oligomerisation. Analytical size exclusion chromatography (SEC) is currently the standard separation technique to quantify protein oligomers. SEC, which very often is also the last step of protein purification, separates molecules according to their hydrodynamic size, often defined by their Stokes or hydrodynamic radius [38], with larger sized molecular species (which are not necessarily larger molecular mass species) eluting before smaller ones. Recent developments of the technique have increased the rapidity of elution, through column parallelization and injection interlacing [39] and/or the use of the latest SEC columns with smaller pore size, allowing improved resolution with smaller bed volumes, reduced elution times (below 10 min) and low sample consumption (5 μg in 20 μl) [40-42]. This should encourage people to resort to SEC as a systematic approach to analyse sample heterogeneity. Aggregates, contaminants and potentially different molecular arrangements of the protein of interest can be readily separated and quantified, with classical online UV detection. One should however keep in mind the fact that the protein sample will be diluted during SEC by as much as a 10-fold factor, which might alter equilibria between oligomeric species.

Furthermore, however “inert” may the gel filtration resins be, some proteins do interact with them, rendering SEC impossible. Two column-free separation techniques may be used as alternatives: asymmetric flow-field flow fractionation (AFFFF), which is also well suited for large molecular assemblies that may be dissociated by SEC [42,43], and capillary electrophoresis with electrophoretic mobility separation (CZE) [22].

##### Static light scattering

Contrary to a widespread belief, the molecular mass of the species eluted in each SEC peak cannot be obtained through column calibration approaches, in which protein standards are separated according to their hydrodynamic radius and not their molecular mass (the correlation between both parameters being far from linear, especially for non-globular and intrinsically disordered proteins). To obtain information about mass, it is necessary to resort to a static light scattering (SLS) detector [44], in combination with a UV or a refractive index (RI) detector. Of note, as in the case of DLS, SLS is also able to detect small amounts of aggregates with high sensitivity, as the light scattering signal is proportional to molecular mass [45]. In size exclusion chromatography with on-line static laser light scattering (SEC-SLS), experimentally determined molecular mass is independent of the elution volume of the protein. Both the total scattered light intensity (which depends on molecular mass and concentration) and the concentration of the protein (using the UV or RI detector) are measured and analysed to determine the molecular mass of the protein as it elutes from the chromatographic column. SEC-SLS is applicable and quite accurate over a broad range of molecular masses (from a few kDa to several MDa), as long as the column is able to resolve completely the different species present in the sample, allowing the area of each peak to be integrated. In order to improve the separation of peaks with respect to traditional SEC, one can resort to ultra-high performance liquid chromatography (UHPLC) systems, which have very recently been made amenable to SLS. As an alternative, AFFF can also be used in conjunction with SLS [42,43].

#### Activity

##### Active protein concentration determination

Once the homogeneity of the protein of interest has been assessed, one has to ensure it is active and functional (Figure 1). An infinite variety of generic or protein-specific functional assays has been designed, relying principally on catalytic and binding properties. An attempt at listing such assays would go much beyond the scope of this review. Efficient assays allow to measure precisely the active concentration of the protein sample, and thus to determine (if the total protein concentration is known: see Total protein concentration determination section) the percentage of purified protein that is indeed functional. One should not overlook such active protein concentration determinations, as it can unfortunately often be found that the proportion of purified protein which is indeed in a native active state is low. This can be due to misfolding issues, to the inability of the protein to reach its native structural state spontaneously or to interferences of sequence additions (such as tags or extra amino acids originating from cloning vectors). But in most cases, this is due to poor (and overlooked) micro-integrity and homogeneity of the purified protein (see Purity and integrity section).

Surface plasmon resonance (SPR) is a convenient technique to determine the active concentration of binding proteins. This is done by exploiting the properties of diffusion of molecules in continuous flow microfluidic devices [46,47]. The so-called “calibration-free concentration analysis” (CFCA) method, which has been implemented in a user-friendly format in different SPR instruments available commercially [48], allows to determine the concentration of protein able to recognize a specific ligand (or protein partner) tethered on a surface. For CFCA measurements, the ligand has to be immobilized at high densities, creating conditions in which the interaction rate of the protein is limited by its diffusion towards the surface (mass transport limitation), and becomes proportional to its active concentration [46,47].

Alternatively, if the protein of interest is tagged, one can resort to a “sandwich” SPR assay to determine directly what proportion of protein is active: a measurable amount of protein is first captured through its tag on a surface on which a tag-specific receptor is immobilized (NTA for His-tag, or an antibody for others) and then titrated by a saturating amount of specific ligand [49].

##### Total protein concentration determination

Different methods are available to measure the total protein concentration in a sample, allowing to deduce the percentage of active protein (see Active protein concentration determination section). Bradford, bicinchonic acid (BCA) and Lowry assays use standards for calibration, which can be a source of error as the composition of the protein of interest may not necessarily match that of the protein standards [26]. It is also possible to use UV-visible absorbance measurements to determine the total protein concentration as long as its extinction coefficient is reliably known or calculated [26,50]. The extinction coefficient at 280 nm is most frequently calculated from the amino acid composition [25], allowing to determine concentrations from UV absorbance at this wavelength (see [26,50] for protocols). However, one should always monitor wider absorbance spectra (at least from 240 to 350 nm), as these can provide much more information than concentration, as already detailed in the two sections referring to UV-visible spectroscopy above.

However, UV absorbance measurements are only usable for concentration determination if the sequence of the protein of interest contains a known amount of tryptophans and tyrosines, the two principal light-absorbing amino acids. If this is not the case, an alternative is to use Fourier Transform Infrared Spectroscopy (FTIR) as initially suggest by Etzion et al. [51]. After subtracting the contribution of water between 1700 nm and 2300 nm, the analysis of the amide band I and II of the IR absorbance spectrum can be used to calculate protein concentration by determining the concentration of amine bonds. Recently, commercially available FTIR equipment has been developed (Direct Detect from Merck Millipore), applying this method to protein samples that are dried on a membrane. The only limitations of the equipment are the minimal and maximal concentrations that can be used (0.2 to 5 mg/ml) and the incompatibility of several amine-containing buffers (HEPES ≥ 25 mM, Tris ≥ 50 mM, …) or additives (EDTA ≥ 10 mM, …). Another alternative is amino acid analysis (AAA) which is a very valuable technique both for protein identification and quantification [52]. Briefly, quantitative AAA involves hydrolyzing the peptide bonds to free individual amino acids, which are then separated, detected and quantified, using purified amino acids as standards (see [52] for protocol).

Nonetheless, UV-visible spectroscopy remains beyond any doubt the most widely spread, cost- and time-efficient technique for total protein concentration determination. To take full advantage of this technique even in the absence of tyrosine and tryptophan residues, one solution can be to use FTIR-based protein quantification and AAA measurements at first, to generate concentration calibration curves for the protein of interest in correlation with UV absorbance (at 280 nm or another wavelength). These calibration curves can then be used to determine the concentration of subsequent samples directly by UV absorbance spectroscopy.

### Optimization, stability and reproducibility of protein samples

Identifying conditions in which a protein sample is “well-behaved” and meets all the required criteria described in Initial sample assessment section is generally not a trivial task. In this section, we aim at providing an overview of potential solutions to overcome difficulties that may arise along the quality control work-flow (Figure 1). We also discuss how to determine optimal conditions for the preservation of good quality samples, and how to ensure that the protein production/purification process that one has devised leads reproducibly to samples of equivalent high quality.

#### Optimization of purity and integrity

A variety of solutions are available to overcome issues of contamination of protein samples with impurities, degradation products or undesired chemically-modified proteins [53]. These go from changing the purification protocols (modifying the washing and elution conditions from affinity chromatography columns, or adding purification steps such as ion-exchange chromatography) to more upstream changes such as the addition of different sets of protease inhibitors, the modification of the conditions of induction of protein expression, the choice of another cloning vector (with a different tag, or a tag placed at another position or at both ends), or even resorting to another expression host system.

#### Optimization of homogeneity and solubility

To remove protein aggregates, it is important to ensure that the last step of the purification process always is size-exclusion chromatography. A column should be chosen that allows elution of the protein of interest well away from the void volume, and thus total separation from large protein aggregates. People often need to concentrate their protein samples in order to attain concentrations high enough for their downstream applications: unfortunately, this process, which resorts to spin concentrators or precipitation/resolubilisation protocols, very frequently tends to induce aggregation. Therefore, one should be careful not to concentrate their sample more than strictly necessarily (avoiding overly high concentrations): this should either be done *before* the final size-exclusion chromatography step, or be followed by an analytical SEC or DLS on part of the concentrated sample to ensure that it has remained free from aggregates.

To minimize the formation of protein aggregates (and to improve solubility), a variety of changes can be made upstream to the production/purification protocol [54]. Adjustment of several parameters of the sample buffer composition (pH, salinity, presence of additives, co-factors or ligands, …) can also dramatically increase homogeneity. People often rely for this on empirical rules that they have learnt with experience, as there is no clear correlation between the stability of a protein and its intrinsic properties (amino acid composition, isoelectric point, secondary structure elements, …). Recent DLS instrumental developments, that allow to process a large number of samples in a 96, 384 or 1536 well plate format, have made buffer condition screening an easy task. Many groups have used DLS as a technique to improve the solubilisation conditions of their proteins, in particular before crystallization studies [55,56]. Buffer matrices for multi-parametric screening of pH, salinity, buffer nature, additives and co-factors can be generated by hand or using simple robotics [57]. Typically samples, at a concentration of 10 mg/ml for a 10 kDa protein or 1 mg/ml for a 100 kDa protein, are diluted 10 times in each test buffer with a consumption of only 2 μl of sample per condition. The homogeneity of the sample and the presence of aggregates (and high-order physiologically irrelevant oligomers) can be monitored in each condition, allowing to select the optimal buffer composition for protein homogeneity.

#### Optimization of protein sample stability and storage

Preservation of good quality protein samples over time is all important, as very often one will not consume all of a sample straight away. People most often rely on hearsay for the short-term or long-term storage of their precious protein samples. A very widely spread belief is that flash freezing (with or without cryoprotectants such as glycerol) is the best method for long-term retention of protein properties. However, this is far from being a general truth, especially because significant denaturation, aggregation and precipitation can occur upon freezing/thawing [58]. Proteins may become unstable and lose their biological activity through a variety of physical or chemical mechanisms, even at cold temperatures [59-61]. The best storage conditions are very much protein-dependent, and may vary from unfrozen aqueous solutions to salted precipitates or freeze-dried solids [59-61].

A practical way to approach this issue is to start by monitoring the time stability of one’s protein sample at a few relevant temperatures (e.g. 4 and 25°C) using DLS and a functional assay, in the optimal buffer for sample homogeneity and solubility (see Optimization of homogeneity and solubility section). Indeed, one may quite often realize this way that simple storage of the protein sample without further processing (for instance at 4°C) provides long enough stability for all down-stream experiments.

Many people also evaluate the thermal stability of their proteins in different buffers, using methods such as differential scanning fluorimetry (DSF, also known as thermal-shift assay) [57]: however, there is no clear correlation between thermodynamic and time stability of a protein, and it is therefore not straightforward to obtain insight about the long-term stability of a sample from its thermal stability analysis. On the contrary, thermodynamic stability generally correlates with rigidity [62], which is of particular importance when the downstream application is structural characterization (for instance by X-ray crystallography).

If a protein needs to be stored for an undetermined period, one can explore different methods (freezing with or without cryoprotectants, lyophilization,… [59-61]) and determine their effect on the properties of the sample using DLS and a functional assay. Of note, the best storage conditions may be largely different from the experimental conditions for downstream applications, so a preliminary desalting or dialysis might be needed before quality control.

#### Determination of protein sample reproducibility and lot-to-lot consistency

A fundamental principle of good laboratory practices is that experiments need to be reproduced and should thus be reproducible, both within a laboratory and between research groups. During the lifetime of a project, it is therefore very likely that one will need to prepare more than a single sample of a given protein. Other groups might also need to prepare it independently in the frame of collaborations or comparability studies. Determining the robustness of one’s production/purification process and its capacity to reproducibly deliver samples of equivalent quality is therefore all-important. However, once the quality of a purified protein sample has been fully assessed and optimized a first time, verification of lot-to-lot consistency does not necessarily require the repetition of the whole quality control work-flow (Figure 1B).

A very practical way to rapidly estimate the equivalence of protein lots is to verify the conformity of their “spectral signatures”. The most straightforward is to compare UV-visible spectra which, as has been stressed above, contain a wealth of information beyond simple 280 nm absorbance. This may be profitably complemented by circular dichroism (CD) in the far-UV, which provides information about the global content of secondary structure elements in a protein [63,64]. Of note, contrary to a widespread belief, the presence of secondary structure elements in a protein (“foldedness”) is not by itself a quality control criterium, especially as many proteins are either intrinsically disordered or contain unfolded segments in their native state. But differences between the CD spectra acquired for two different lots of the same protein (in the same buffer) may readily reveal divergences in folding that could correlate with differences in active concentration, especially if spectral similarity is analysed quantitatively rather than visually [65,66].

“Thermal denaturation signatures”, determined by techniques such as CD or differential scanning calorimetry (DSC, [67]), can also be a very convenient and accurate way to determine the equivalence of protein lots, provided special attention is given to the equivalence of protein sample conditioning buffers. Indeed, differences between protein lots can translate into detectable differences in the global shape of their denaturation profiles [68].

Apart from spectral and thermal denaturation signatures, MS (for integrity), DLS (for homogeneity), analytical SEC (for both purity and homogeneity) and a functional assay are the most convenient and discriminating methods to assess the reproducibility and equivalence in quality of distinct protein lots.

## Conclusion

In this review, we have attempted to cover all the aspects of protein quality control, from the necessary initial sample assessment to sample optimization. For each step, a set of relevant techniques has been suggested (Figure 1A). The first-line methods are essential and should be used systematically for a full quality control assessment. Different complementary methods can be added depending on the protein sample peculiarities and quality control requirements. The suggested approaches for first line assessment include the “basic requirements for evaluating protein quality” that have been recently proposed [10], but go significantly beyond them. We also suggest a sequential experimental work-flow, to be followed as a check-list in order to optimize the time and effort spent on each sample (Figure 1B). This work-flow elaborates the protein quality control and storage optimization steps of the general protein production/purification pipeline [10]. Overall, this global synthetic step-by-step overview should hopefully lead to better protein samples and therefore to better chances of success in downstream applications. In line with community-based efforts that have been deployed in other fields like structural biology [69,70], proteomics and interactomics [71-74] or quantitative real-time PCR [75,76], research relying on purified proteins would gain significant reliability and credibility from the implementation of good practices, such as the systematic and transparent reporting of the results of purified protein quality control assessments, at least in the supplementary information sections of scientific publications.

## Abbreviations

SDS–PAGE: Sodium dodecyl sulfate polyAcrylamide gel electrophoresis
MS: Mass spectrometry
MALDI: Matrix-assisted laser desorption/ionization
IEF: Iso-electric focusing
CE: Capillary electrophoresis
DLS: Dynamic light Scattering
SEC: Size Exclusion Chromatography
AFFF: Asymmetric Flow-Field flow fractionation
RI: Refractive index
SLS: Static light scattering
SPR: Surface plasmon resonance
CFCA: Calibration-Free Concentration Analysis
FTIR: Fourier Transform Infrared Spectroscopy
AAA: Amino acid analysis
CD: Circular dichroism
